# Entropy analysis for a novel peristaltic flow in a curved heated endoscope: an application of applied sciences

**DOI:** 10.1038/s41598-023-28047-8

**Published:** 2023-01-27

**Authors:** Sohail Nadeem, Salman Akhtar, Anber Saleem, Nevzat Akkurt, Shahah Almutairi, Hassan Ali Ghazwani, Sayed M. Eldin

**Affiliations:** 1 Department of Mathematics, Quaid-i-Azam University 45320, Islamabad, 44000 Pakistan; 2 Department of Mathematics, Wenzhou University, Wenzhou, 325035 China; 3 Department of Anatomy, School of Dentistry, SZABMU, Islamabad, Pakistan; 4 Department of Mathematics, Quaid-i-Azam University, 62000 Islamabad, Pakistan; 5 Mathematics Department, Faculty of Sciences, Northern Border University, Arar, 1321 Saudi Arabia; 6 Department of Mechanical Engineering, Faculty of Engineering, Jazan University, P.O box 45124, Jazan, Kingdom of Saudi Arabia; 7 Center of Research, Faculty of Engineering, Future University in Egypt, New Cairo, 11835 Egypt

**Keywords:** Biophysics, Mathematics and computing

## Abstract

Entropy interpretation with a descriptive heat generation analysis is carried out for the heated flow between two homocentric and sinusoidally fluctuating curved tubes. A novel peristaltic endoscope is considered for the first time inside a curved tube with evaluation of heat transfer and entropy. This flexible and novel endoscope with peristaltic locomotion is more efficient for endoscopy of complex mechanical structures and it is more comfortable for patients undergoing the endoscopy of a human organs. A comprehensive mathematical model is developed that also completely evaluates the heat transfer analysis for this novel endoscope. Certain and systematic computations are performed with the help of Mathematica software and exact mathematical as well as graphical solutions are obtained. Entropy has a lower rate that is almost zero entropy in the central region of these two curved tubes, but maximum entropy is noted near the sinusoidally deformable walls of both the endoscope and channel.

## Introduction

Endoscopes are important due to their immense number of medical as well as industrial applications. The complex structures of distinct big machineries like large cruise ship engines, aircrafts, various engineering appliances and endoscopy of human organs are the major applications. A novel endoscope was developed by Mangan et al.^1^, that is basically a locomotive endoscope which can turn through sharp edges and complex curved structures etc. This is known as a peristaltic endoscope. The peristaltic locomotion of this efficient endoscope has made a tremendous advancement in the field of endoscopy with an increased number of engineering and medical applications. Misiery et al.^2^ had disclosed the theoretical study of peristaltic motion inside a tube with insertion of an endoscope. Tripathi^3^ had interpreted the numerical study of a non-Newtonian flow inside a tube with detailed and comprehensive applications of endoscopy. Further, some recent and relevant references that evaluate the peristaltic flow with endoscopic applications are given^4–10^.

The heat generation analysis with peristaltic flow and endoscopic applications is also evaluated by many recent researchers. Mekheimer^11^ had provided the heat transfer study of a Newtonian flow inside an annulus with applications of an endoscope. Nadeem et al.^12^ had numerically interpreted the important characteristics of heat transfer for an endoscope. Irshad et al.^13^ had mathematically modelled the heat transfer for peristaltic flow inside a curved channel with endoscopic applications. Shahzadi et al.^14^ had disclosed the mathematical analysis of heat transfer inside a curved annulus with endoscopic applications. Entropy generation is also important in many of the endoscopic applications. The analysis of entropy also clearly indicates that how much disorder or disturbance is caused by the moving endoscope, whether it is endoscopy of human organs or complex mechanical structures etc. Narla et al.^15^ had provided a detailed model that interprets the entropy with heat transfer analysis for the peristaltic flow inside a curved channel. Moreover, the relevant research articles that provide the theoretical interpretation of heat transfer and entropy generation for the flow inside a curved tube are referred as^16–23^.

This research work discloses a mathematical study of entropy interpretation with heat generation for a novel peristaltic endoscope. We have considered a heated Newtonian flow between these two sinusoidally fluctuating curved tubes having same centre. The inner wall of this peristaltic endoscope is sinusoidally deforming and the outer wall of this tube having this endoscope is also sinusoidally deforming wall. This is extension work of the study provided in^24^ with heat transfer and thorough entropy interpretation. The equations that interpret this problem are evaluated with the help of Mathematica software and we have availed exact solutions for temperature, entropy, velocity and pressure gradient etc. The graphical solutions are also presented that are in complete harmony with the mathematical computations.

## Mathematical model

**Figure 1:**
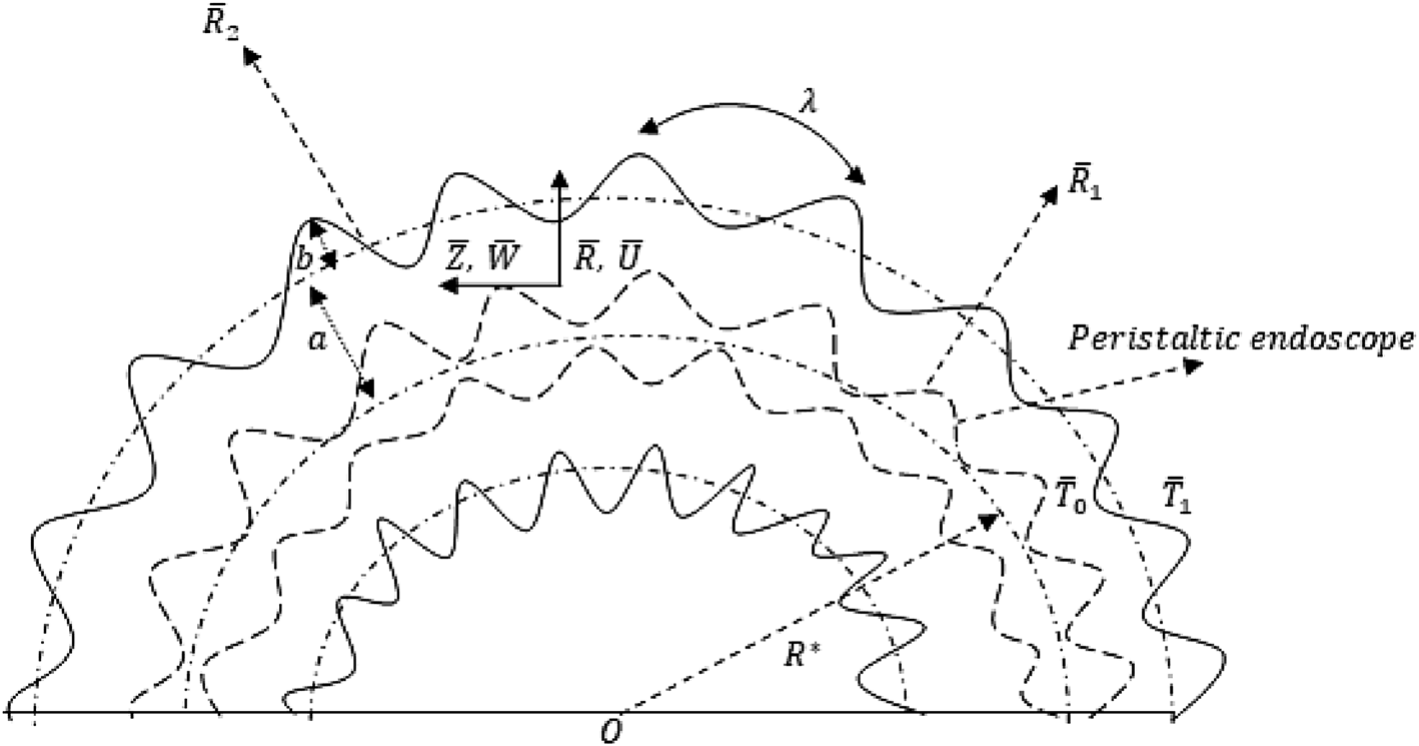
Geometrical model of the problem.

A heated, viscous Newtonian flow is considered between two sinusoidally fluctuating homocentric curved tubes. The inner curved tube is a novel peristaltic endoscope, and the geometrical model is given by Fig. 1 as follows

The sinusoidally fluctuating external wall of the curved tube as well as the internal sinusoidally fluctuating wall of the novel peristaltic endoscope is mathematically considered by following equations given as^15–17,24^

1 $$\begin{gathered} \overline{R}_{2} = a + bSin\frac{2\pi }{\lambda }\left( {\overline{Z} - c\overline{t}} \right), \hfill \\ \overline{R}_{1} = n\overline{R}_{2} = na + nbSin\frac{2\pi }{\lambda }\left( {\overline{Z} - c\overline{t}} \right), \hfill \\ \end{gathered}$$

where $"n"$ has a range between 0 and 1.

The dimensional representation of mathematical equations is narrated as follows^15–17,24^

2 $$\frac{\partial }{\partial \overline{R} }\left((\overline{R }+{R}^{*})\overline{U }\right)+{R}^{*}\frac{\partial \overline{W} }{\partial \overline{Z} }=0,$$

3 $$\begin{gathered} \rho \left( {\frac{{\partial \overline{U}}}{{\partial \overline{t}}} + \overline{U}\frac{{\partial \overline{U}}}{{\partial \overline{R}}} + \frac{{R^{*} \overline{W}}}{{\overline{R} + R^{*} }}\frac{{\partial \overline{U}}}{{\partial \overline{Z}}} - \frac{{\overline{W}^{2} }}{{\overline{R} + R^{*} }}} \right) = - \frac{{\partial \overline{P}}}{{\partial \overline{R}}} \hfill \\ \;\; + \mu \left[ {\frac{1}{{\left( {\overline{R} + R^{*} } \right)}}\frac{\partial }{{\partial \overline{R}}}\left\{ {\left( {R^{*} + \overline{R}} \right)\frac{{\partial \overline{U}}}{{\partial \overline{R}}}} \right\} + \left( {\frac{{R^{*} }}{{R^{*} + \overline{R}}}} \right)^{2} \frac{{\partial^{2} \overline{U}}}{{\partial \overline{Z}^{2} }} - \frac{{\overline{U}}}{{\left( {R^{*} + \overline{R}} \right)^{2} }} - \frac{{2R^{*} }}{{\left( {R^{*} + \overline{R}} \right)^{2} }}\frac{{\partial \overline{W}}}{{\partial \overline{Z}}}} \right] \hfill \\ \end{gathered}$$

4 $$\begin{gathered} \rho \left( {\frac{{\partial \overline{W}}}{{\partial \overline{t}}} + \overline{U}\frac{{\partial \overline{W}}}{{\partial \overline{R}}} + \frac{{R^{*} \overline{W}}}{{\overline{R} + R^{*} }}\frac{{\partial \overline{W}}}{{\partial \overline{Z}}} + \frac{{\overline{W}\overline{U}}}{{\overline{R} + R^{*} }}} \right) = - \left( {\frac{{R^{*} }}{{\overline{R} + R^{*} }}} \right)\frac{{\partial \overline{P}}}{{\partial \overline{Z}}} \hfill \\ \;\;\; + \mu \left[ {\frac{1}{{\left( {\overline{R} + R^{*} } \right)}}\frac{\partial }{{\partial \overline{R}}}\left\{ {\left( {R^{*} + \overline{R}} \right)\frac{{\partial \overline{W}}}{{\partial \overline{R}}}} \right\} + \left( {\frac{{R^{*} }}{{R^{*} + \overline{R}}}} \right)^{2} \frac{{\partial^{2} \overline{W}}}{{\partial \overline{Z}^{2} }} - \frac{{\overline{W}}}{{\left( {R^{*} + \overline{R}} \right)^{2} }} + \frac{{2R^{*} }}{{\left( {R^{*} + \overline{R}} \right)^{2} }}\frac{{\partial \overline{U}}}{{\partial \overline{Z}}}} \right], \hfill \\ \end{gathered}$$

5 $$\rho {C}_{p}\left(\frac{\partial \overline{T} }{\partial \overline{t} }+\frac{{R}^{*}\overline{W} }{(\overline{R }+{R}^{*})}\frac{\partial \overline{T} }{\partial \overline{Z} }+\overline{U }\frac{\partial \overline{T} }{\partial \overline{R} }\right)=k\left[{\left(\frac{{R}^{*}}{\overline{R }+{R}^{*}}\right)}^{2}\frac{{\partial }^{2}\overline{T} }{\partial {\overline{Z} }^{2}}+\frac{1}{\overline{R }+{R}^{*}}\frac{\partial \overline{T} }{\partial \overline{R} }+\frac{{\partial }^{2}\overline{T} }{\partial {\overline{R} }^{2}}\right]+\mu \left[2\left\{{\left(\frac{\partial \overline{U} }{\partial \overline{R} }\right)}^{2}+{\left(\frac{{R}^{*}}{{R}^{*}+\overline{R} }\frac{\partial \overline{W} }{\partial \overline{Z} }+\frac{\overline{U} }{\overline{R }+{R}^{*}}\right)}^{2}\right\}+{\left(\frac{\partial \overline{W} }{\partial \overline{R} }+\frac{{R}^{*}}{{R}^{*}+\overline{R} }\frac{\partial \overline{U} }{\partial \overline{Z} }-\frac{\overline{W} }{\overline{R }+{R}^{*}}\right)}^{2}\right],$$

The mathematical equations that primarily connect the two separate reference frames are narrated as

6 $$\overline{z }=\overline{Z }-c\overline{t }, \overline{r }=\overline{R }, \overline{p }=\overline{P } ,\overline{w }=\overline{W }-c, \overline{u }=\overline{U },$$

The dimensionless terms that have appeared during the simplification of above mathematical equations into non-dimensional form are given as

7 $$\begin{gathered} r = \frac{{\overline{r}}}{a}, z = \frac{{\overline{z}}}{\lambda }, w = \frac{{\overline{w}}}{c}, u = \frac{{\lambda \overline{u}}}{ac}, p = \frac{{a^{2} \overline{p}}}{c\lambda \mu }, \beta = \frac{a}{\lambda }, s = \frac{{R^{*} }}{a}, \phi = \frac{b}{a}, r_{1} = \frac{{\overline{R}_{1} }}{a}, r_{2} = \frac{{\overline{R}_{2} }}{a}, \hfill \\ \theta = \frac{{\overline{T} - \overline{T}_{1} }}{{\overline{T}_{0} - \overline{T}_{1} }}, B_{r} = \frac{{\mu c^{2} }}{{k\left( {\overline{T}_{0} - \overline{T}_{1} } \right)}}, S = \frac{{\overline{S}}}{{{\raise0.7ex\hbox{${k\left( {\overline{T}_{0} - \overline{T}_{1} } \right)^{2} }$} \!\mathord{\left/ {\vphantom {{k\left( {\overline{T}_{0} - \overline{T}_{1} } \right)^{2} } {\overline{T}_{1}^{2} a^{2} }}}\right.\kern-0pt} \!\lower0.7ex\hbox{${\overline{T}_{1}^{2} a^{2} }$}}}}, \Omega = \frac{{\overline{T}_{0} - \overline{T}_{1} }}{{\overline{T}_{1} }}, \hfill \\ \end{gathered}$$

The dimensionless and eventual simplified form of above mathematical equations after utilizing the theoretical approximation of λ $\to \infty$ is provided as

8 $$\frac{\partial p}{\partial r}=0,$$

9 $$\left(\frac{s}{r+s}\right)\frac{\partial p}{\partial z}=\frac{1}{(r+s)}\frac{\partial }{\partial r}\left\{(s+r)\frac{\partial w}{\partial r}\right\}-\frac{(w+1)}{{(s+r)}^{2}},$$

10 $$\frac{{\partial }^{2}\theta }{\partial {r}^{2}}+\frac{1}{(r+s)}\frac{\partial \theta }{\partial r}+{B}_{r}{\left(\frac{\partial w}{\partial r}-\frac{(w+1)}{(r+s)}\right)}^{2}=0,$$

The pertinent boundary conditions are

11 $$\begin{aligned} w & = - 1\;{\text{at}}\;r = r_{1} = n + n\phi Sin\left( {2\pi z} \right), \\ w & = - 1\;{\text{at}}\;r = r_{2} = 1 + \phi Sin\left( {2\pi z} \right), \\ \end{aligned}$$

12 $$\begin{gathered} \theta = 1\;{\text{at}}\;r = r_{1} = n + n\phi Sin\left( {2\pi z} \right), \hfill \\ \theta = 0\;{\text{at}}\;r = r_{2} = 1 + \phi Sin\left( {2\pi z} \right), \hfill \\ \end{gathered}$$

### Entropy analysis

The dimensional form of entropy analysis is considered as follows

13 $$\overline{S }=\frac{k}{{\overline{T} }_{1}^{2}}\left[{\left(\frac{{R}^{*}}{\overline{R }+{R}^{*}}\frac{\partial \overline{T} }{\partial \overline{Z} }\right)}^{2}+{\left(\frac{\partial \overline{T} }{\partial \overline{R} }\right)}^{2}\right]+\frac{\mu }{{\overline{T} }_{1}}\left[2\left\{{\left(\frac{\partial \overline{U} }{\partial \overline{R} }\right)}^{2}+{\left(\frac{{R}^{*}}{\overline{R }+{R}^{*}}\frac{\partial \overline{W} }{\partial \overline{Z} }+\frac{\overline{U} }{\overline{R }+{R}^{*}}\right)}^{2}\right\}+{\left(\frac{\partial \overline{W} }{\partial \overline{R} }+\frac{{R}^{*}}{\overline{R }+{R}^{*}}\frac{\partial \overline{U} }{\partial \overline{Z} }-\frac{\overline{W} }{\overline{R }+{R}^{*}}\right)}^{2}\right],$$

After utilizing the given transformations and dimensionless quantities, we avail the following simplest and dimensionless form of entropy that is narrated as

14 $$S={\left(\frac{\partial \theta }{\partial r}\right)}^{2}+\frac{{B}_{r}}{\Omega }{\left(\frac{\partial w}{\partial r}-\frac{w+1}{(r+s)}\right)}^{2},$$

The value of Bejan number is computed as

15 $${B}_{e}=\frac{{S}_{cond.}}{{S}_{cond.}+{S}_{visc.}},$$

After inserting the pertinent values in Eq. (15), we have

16 $${B}_{e}=\frac{{\left(\frac{\partial \theta }{\partial r}\right)}^{2}}{{\left(\frac{\partial \theta }{\partial r}\right)}^{2}+\frac{{B}_{r}}{\Omega }{\left(\frac{\partial w}{\partial r}-\frac{w+1}{(r+s)}\right)}^{2}},$$

### Exact solution

The velocity solution is computed by solving Eq. (9) with Eq. (11) and given as

17 $$w=-1+\frac{\left[\frac{\partial p}{\partial z}s\left\{\left({r}_{1}-{r}_{2}\right){\left(r+s\right)}^{2}\left({r}_{1}+{r}_{2}+2s\right)Log\left(r+s\right)-\left(r-{r}_{2}\right){\left({r}_{1}+s\right)}^{2}\left(r+{r}_{2}+2s\right)Log\left({r}_{1}+s\right)+\left(r-{r}_{1}\right){\left({r}_{2}+s\right)}^{2}\left(r+{r}_{1}+2s\right)Log\left({r}_{2}+s\right)\right\}\right]}{\left(2\left({r}_{1}-{r}_{2}\right)\left(r+s\right)\left({r}_{1}+{r}_{2}+2s\right)\right)}.$$

Further, the rate of volume flow is defined as

18 $$F=\underset{{r}_{1}}{\overset{{r}_{2}}{\int }}r.wdr,$$

The pressure gradient is computed from Eq. (18) and narrated as

19 $$\frac{dp}{dz}=\frac{\left(36\left({r}_{1}-{r}_{2}\right)\left(2F-{r}_{1}^{2}+{r}_{2}^{2}\right)({r}_{1}+{r}_{2}+2s)\right)}{\left[s\left\{{\left({r}_{1}-{r}_{2}\right)}^{2}\left({r}_{1}+{r}_{2}+2s\right)\left(4\left({r}_{1}^{2}+{r}_{1}{r}_{2}+{r}_{2}^{2}\right)+3\left({r}_{1}+{r}_{2}\right)s-6{s}^{2}\right)-12{\left({r}_{1}+s\right)}^{2}{\left({r}_{2}+s\right)}^{2}\left(Log\left({r}_{1}+s\right)-Log\left({r}_{2}+s\right)\right)(2{r}_{1}-2{r}_{2}-3sLog\left({r}_{1}+s\right)+3sLog({r}_{2}+s))\right\}\right]}$$

Moreover, $Q=F+2$ and the pressure rise is numerically computed as

20 $$\Delta P=\underset{0}{\overset{1}{\int }}\frac{\partial p}{\partial z}dz,$$

The temperature solution is calculated by evaluating Eq. (10) with Eq. (12) and narrated as

21 $$\begin{aligned}\theta &=\frac{1}{\left[16{({r}_{1}-{r}_{2})}^{2}{(r+s)}^{2}{({r}_{1}+{r}_{2}+2s)}^{2}\left\{Log\left({r}_{1}+s\right)-Log({r}_{2}+s)\right\}\right]}\\ &\quad\left[8{B}_{r}{\left(\frac{dp}{dz}\right)}^{2}\left({r}_{1}-{r}_{2}\right){s}^{2}{\left(r+s\right)}^{2}{\left({r}_{1}+s\right)}^{2}{\left({r}_{2}+s\right)}^{2}\left({r}_{1}+{r}_{2}+2s\right){(Log(r+s))}^{2}{\left\{Log\left({r}_{1}+s\right)-Log({r}_{2}+s)\right\}}^{2}\right.\\&\quad \left.+{\left({r}_{1}-{r}_{2}\right)}^{2}{\left(r+s\right)}^{2}{\left({r}_{1}+{r}_{2}+2s\right)}^{2}\left\{-16+{B}_{r}{\left(\frac{dp}{dz}\right)}^{2} \left(r-{r}_{1}\right){s}^{2}\left(r+{r}_{1}+2s\right)\right\}Log\left({r}_{2}+s\right)-4{B}_{r}{\left(\frac{dp}{dz}\right)}^{2} \right.\\&\quad \left. \left(r-{r}_{1}\right){s}^{2}{\left({r}_{1}+s\right)}^{2}{\left({r}_{2}+s\right)}^{4}\left(r+{r}_{1}+2s\right){(Log({r}_{2}+s))}^{3}+4{B}_{r}{\left(\frac{dp}{dz}\right)}^{2}{s}^{2}{\left({r}_{1}+s\right)}^{2}{\left({r}_{2}+s\right)}^{2}{(Log({r}_{1}+s))}^{3} \right.\\&\quad \left. \left\{\left(r-{r}_{2}\right){\left({r}_{1}+s\right)}^{2}\left(r+{r}_{2}+2s\right)+2\left({r}_{1}-{r}_{2}\right){\left(r+s\right)}^{2}\left({r}_{1}+{r}_{2}+2s\right)Log({r}_{2}+s)\right\}-4{B}_{r}{\left(\frac{dp}{dz}\right)}^{2}\right.\\&\quad \left.{s}^{2}{\left({r}_{1}+s\right)}^{2}{\left({r}_{2}+s\right)}^{2}{\left(Log\left({r}_{1}+s\right)\right)}^{2}Log\left({r}_{2}+s\right)\left\{2{r}^{2}{r}_{1}^{2}+{r}^{2}{r}_{2}^{2}-3{r}_{1}^{2}{r}_{2}^{2}+2\left(r+{r}_{1}+{r}_{2}\right)\left(2r{r}_{1}+r{r}_{2}-3{r}_{1}{r}_{2}\right)s \right.\right. \\& \quad \left.\left.+\left(3{r}^{2}+8r{r}_{1}-{r}_{1}^{2}+4\left(r-3{r}_{1}\right){r}_{2}-2{r}_{2}^{2}\right){s}^{2}+2\left(3r-{r}_{1}-2{r}_{2}\right){s}^{3}+4\left({r}_{1}-{r}_{2}\right){\left(r+s\right)}^{2}\left({r}_{1}+{r}_{2}+2s\right)Log\left({r}_{2}+s\right)\right\}\right.\\&\quad\left.-\left({r}_{1}-{r}_{2}\right){\left(r+s\right)}^{2}\left({r}_{1}+{r}_{2}+2s\right)Log\left(r+s\right)\left\{-\left({r}_{1}-{r}_{2}\right)\left({r}_{1}+{r}_{2}+2s\right)\left(16+{B}_{r}{\left(\frac{dp}{dz}\right)}^{2}\left({r}_{1}-{r}_{2}\right){s}^{2}\left({r}_{1}+{r}_{2}+2s\right)\right)\right.\right.\\&\quad\left.\left.+4{B}_{r}{\left(\frac{dp}{dz}\right)}^{2}{s}^{2}{\left({r}_{1}+s\right)}^{2}{\left({r}_{2}+s\right)}^{2}{\left(Log\left({r}_{1}+s\right)-Log\left({r}_{2}+s\right)\right)}^{2}\left(1+2Log\left({r}_{1}+s\right)+2Log\left({r}_{2}+s\right)\right)\right\}\right. \\&\quad \left. +{B}_{r}{\left(\frac{dp}{dz}\right)}^{2}{s}^{2}Log({r}_{1}+s)\left\{-\left(r-{r}_{2}\right){\left({r}_{1}-{r}_{2}\right)}^{2}{\left(r+s\right)}^{2}\left(r+{r}_{2}+2s\right){\left({r}_{1}+{r}_{2}+2s\right)}^{2}+4{\left({r}_{1}+s\right)}^{2}{\left({r}_{2}+s\right)}^{2}{(Log({r}_{2}+s))}^{2}\right.\right.\\&\quad \left.\left.\left({r}^{2}{r}_{1}^{2}+2{r}^{2}{r}_{2}^{2}-3{r}_{1}^{2}{r}_{2}^{2}+2\left(r+{r}_{1}+{r}_{2}\right)\left(r{r}_{1}+2r{r}_{2}-3{r}_{1}{r}_{2}\right)s+\left(3{r}^{2}+4r{r}_{1}-2{r}_{1}^{2}+8r{r}_{2}-12{r}_{1}{r}_{2}-{r}_{2}^{2}\right){s}^{2}\right.\right.\right.\\&\quad \left.\left.\left.+2\left(3r-2{r}_{1}-{r}_{2}\right){s}^{3}+2\left({r}_{1}-{r}_{2}\right){\left(r+s\right)}^{2}\left({r}_{1}+{r}_{2}+2s\right)Log({r}_{2}+s)\right)\right\}\right]\end{aligned}$$

### Validation of results

The exact solutions are obtained for this problem by using Mathematica software. The velocity and temperature exact solution profiles exactly satisfy the formulated equations and boundary conditions for this problem. The graphical results provided in this study also show accordance with the formulated problem and conditions. The graphical results also show validation with already published work given in^24^.

## Results and discussion

**Figure 2:**
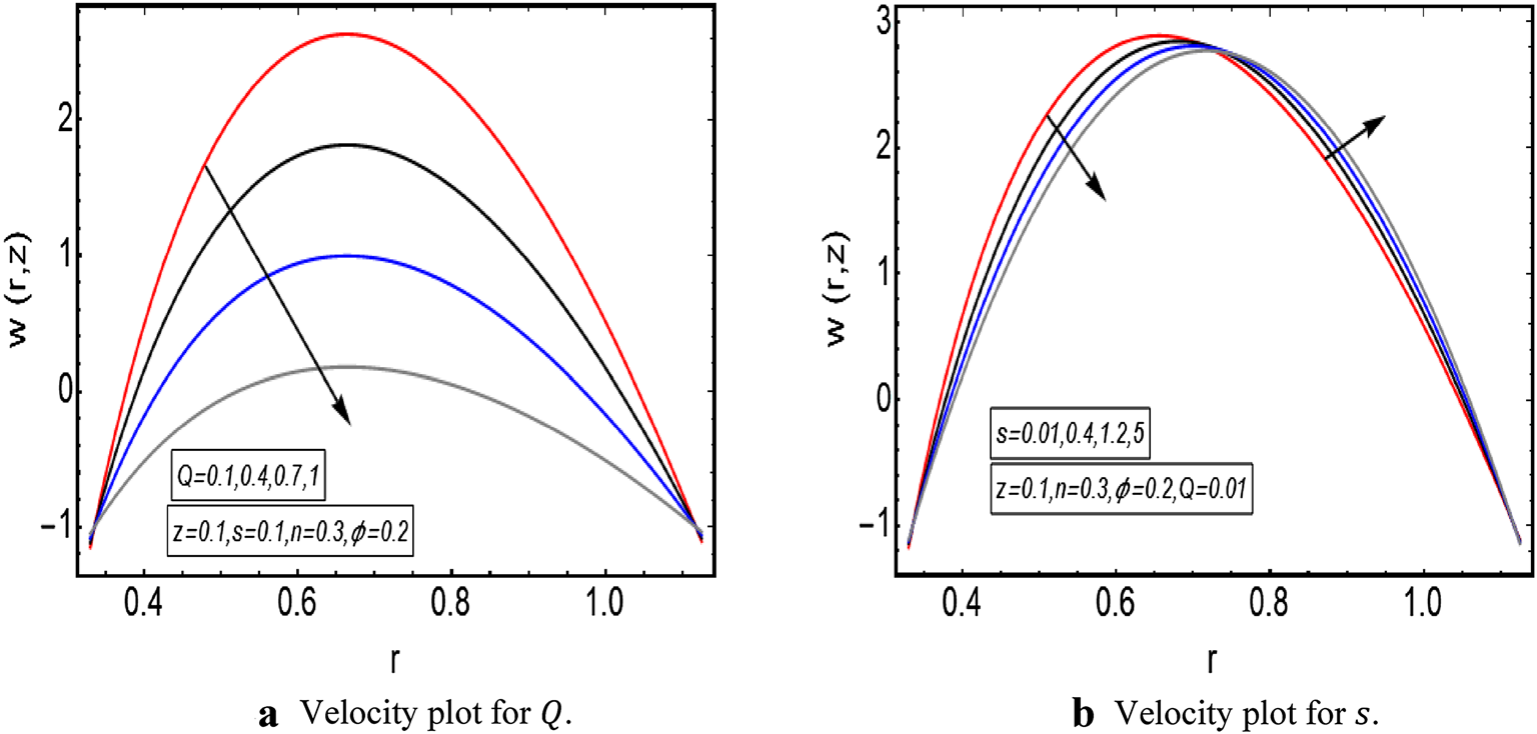
(**a**) Velocity plot for $Q$. (**b**) Velocity plot for $s$.

**Figure 3:**
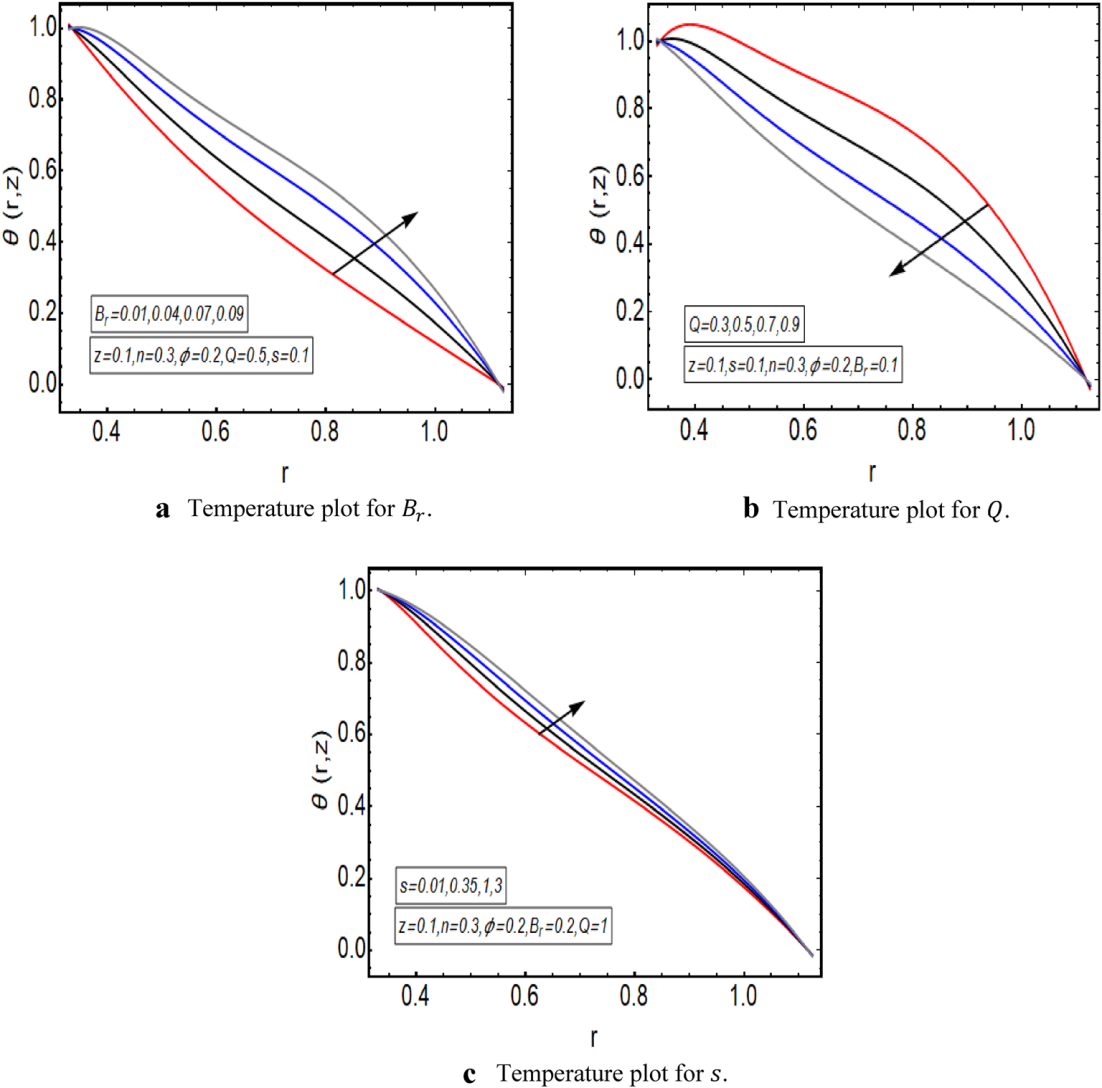
(**a**) Temperature plot for ${B}_{r}$. (**b**) Temperature plot for $Q$. (**c**) Temperature plot for $s$.

**Figure 4:**
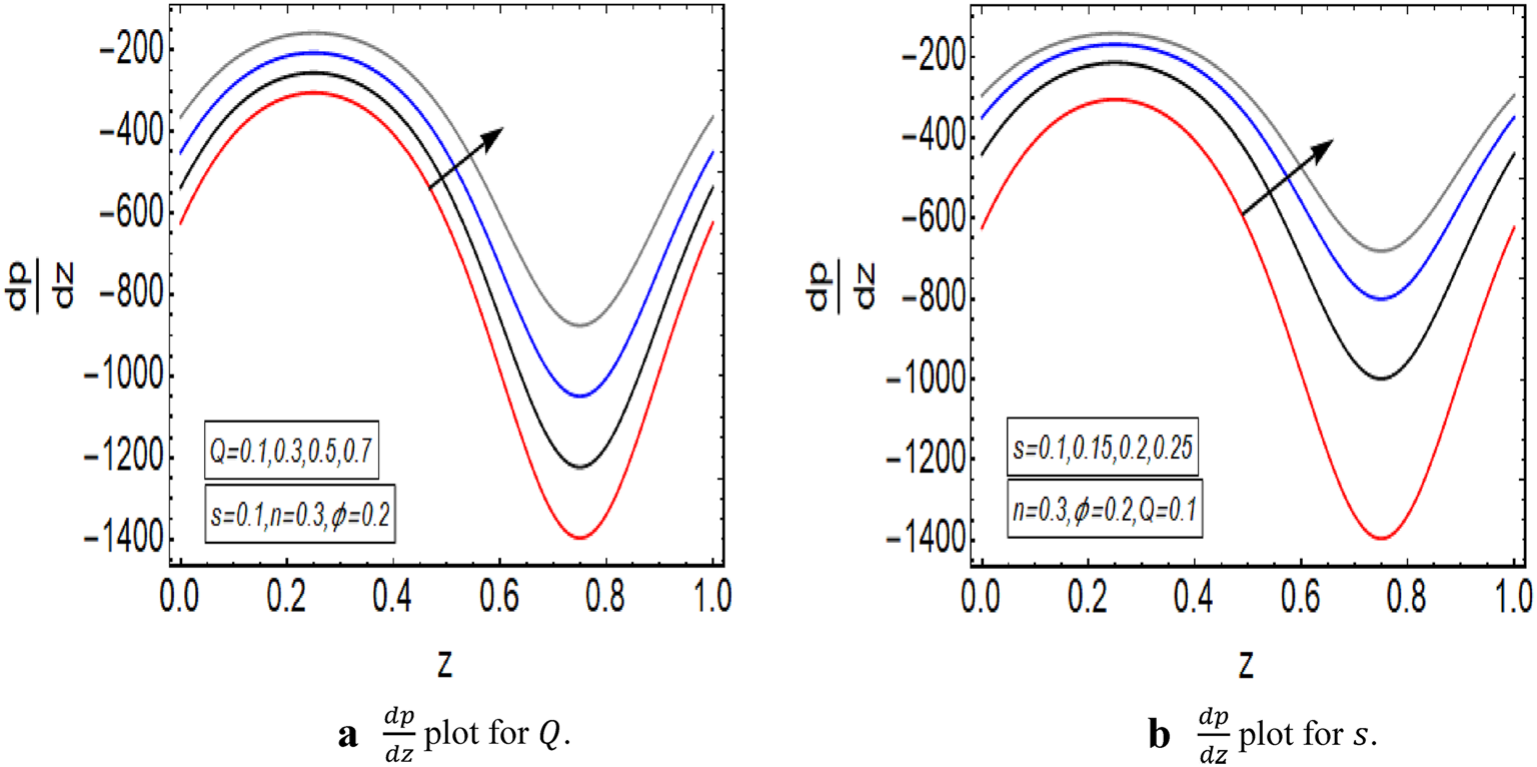
(**a**) $\frac{dp}{dz}$ plot for $Q$. (**b**) $\frac{dp}{dz}$ plot for $s$.

**Figure 5:**
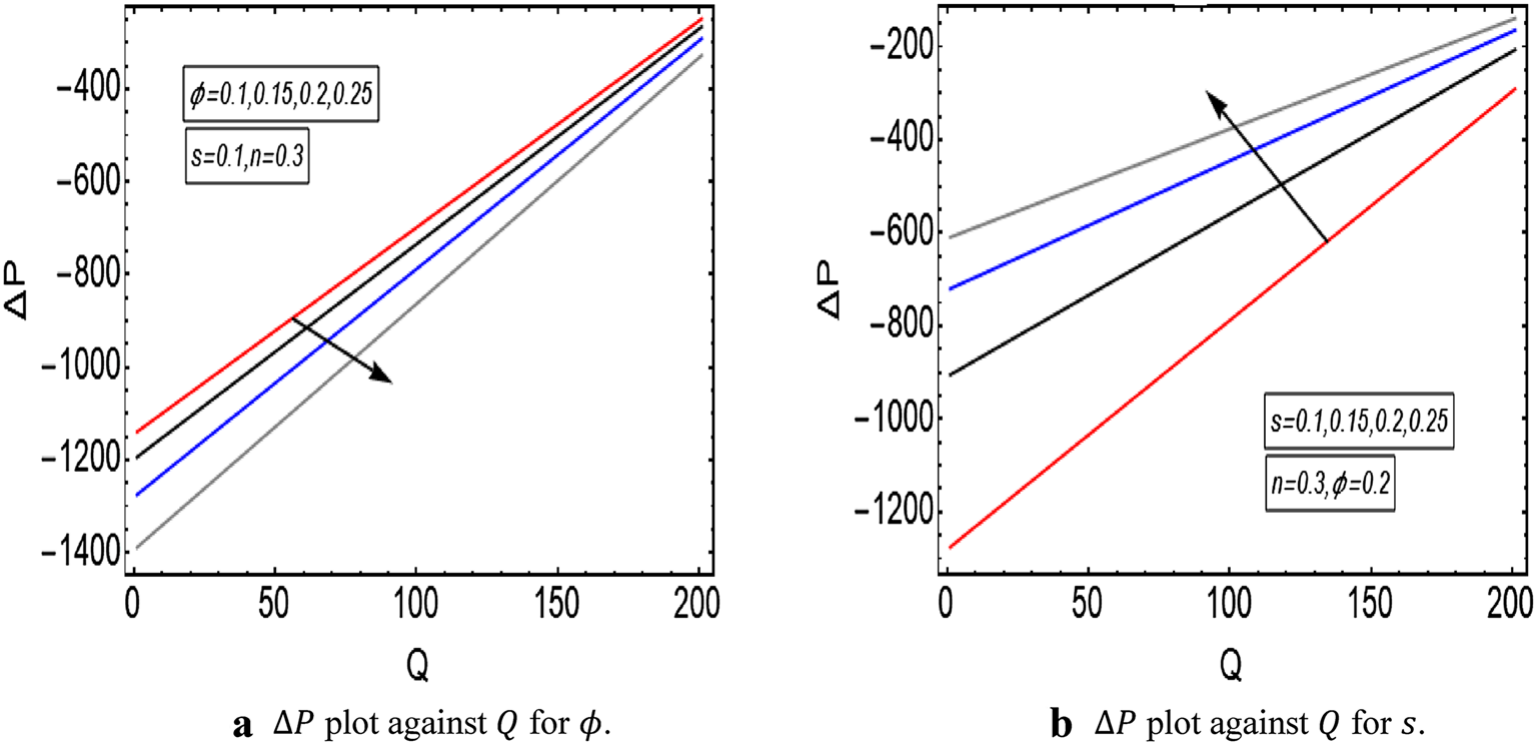
(**a**) $\Delta P$ plot against $Q$ for $\phi$. (**b**) $\Delta P$ plot against $Q$ for $s$.

**Figure 6:**
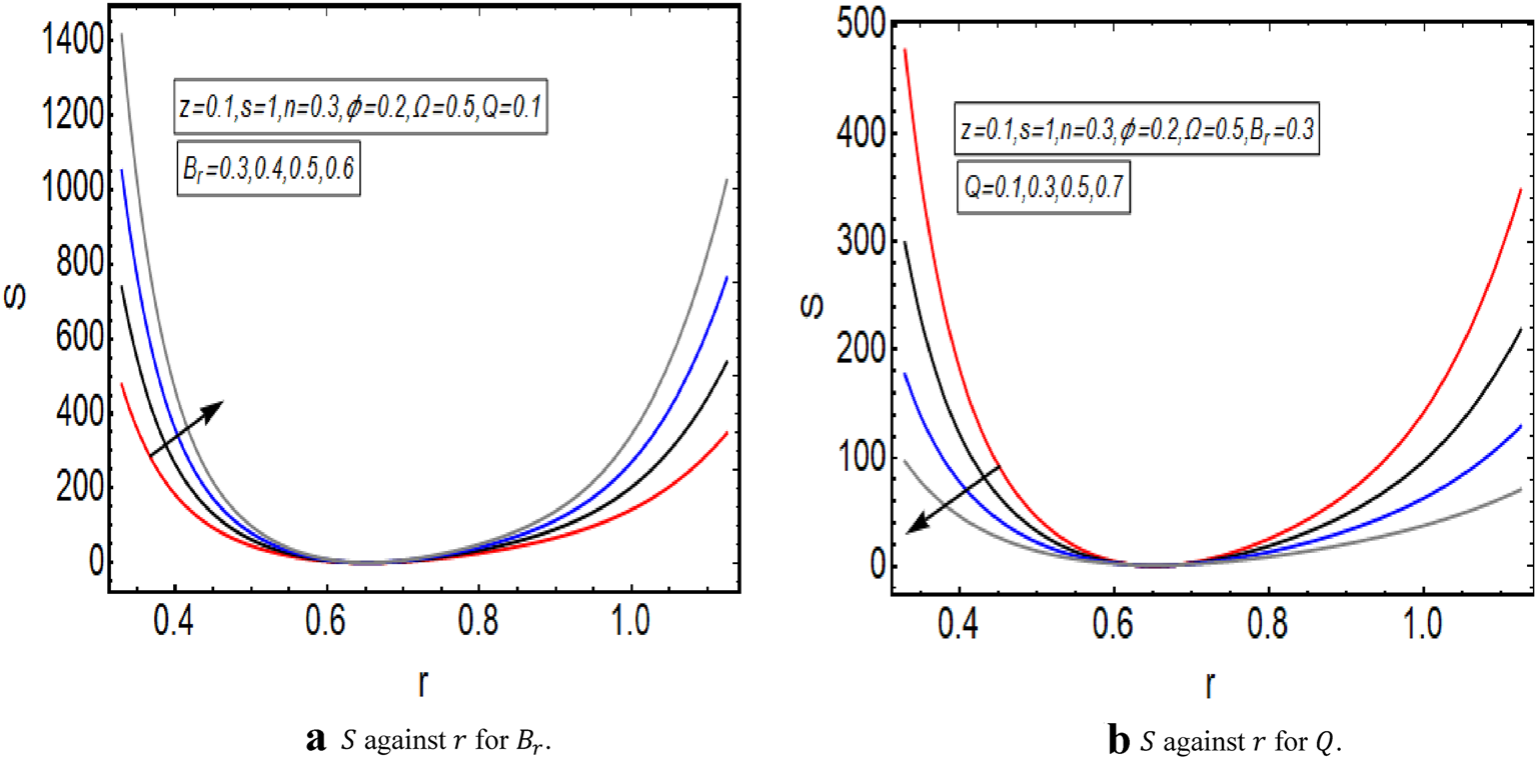
(**a**) $S$ against $r$ for ${B}_{r}$. (**b**) $S$ against $r$ for $Q$.

**Figure 7:**
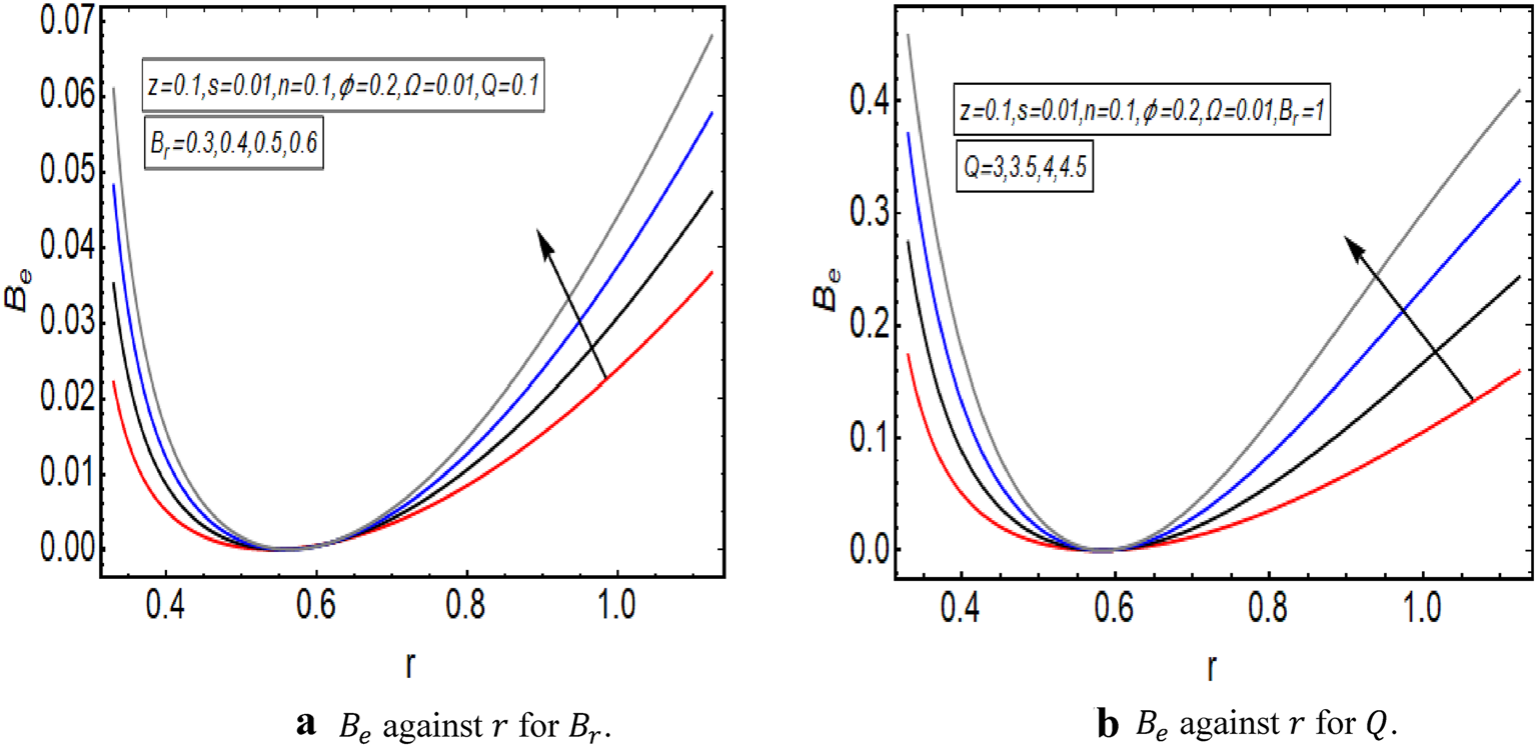
(**a**) ${B}_{e}$ against $r$ for ${B}_{r}$. (**b**) ${B}_{e}$ against $r$ for $Q$.

**Figure 8:**
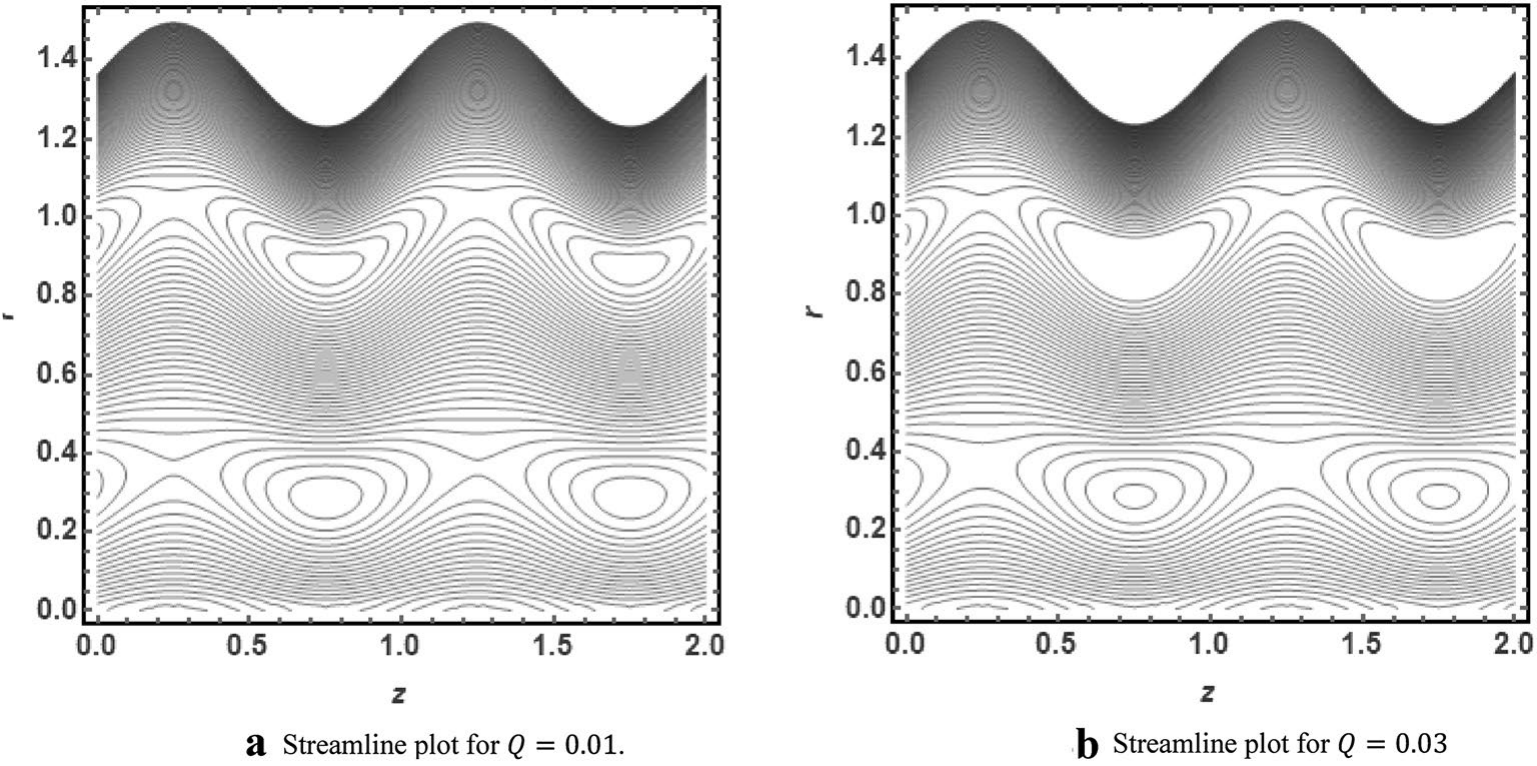
(**a**) Streamline plot for $Q=0.01$. (**b**) Streamline plot for $Q=0.03$.

**Figure 9:**
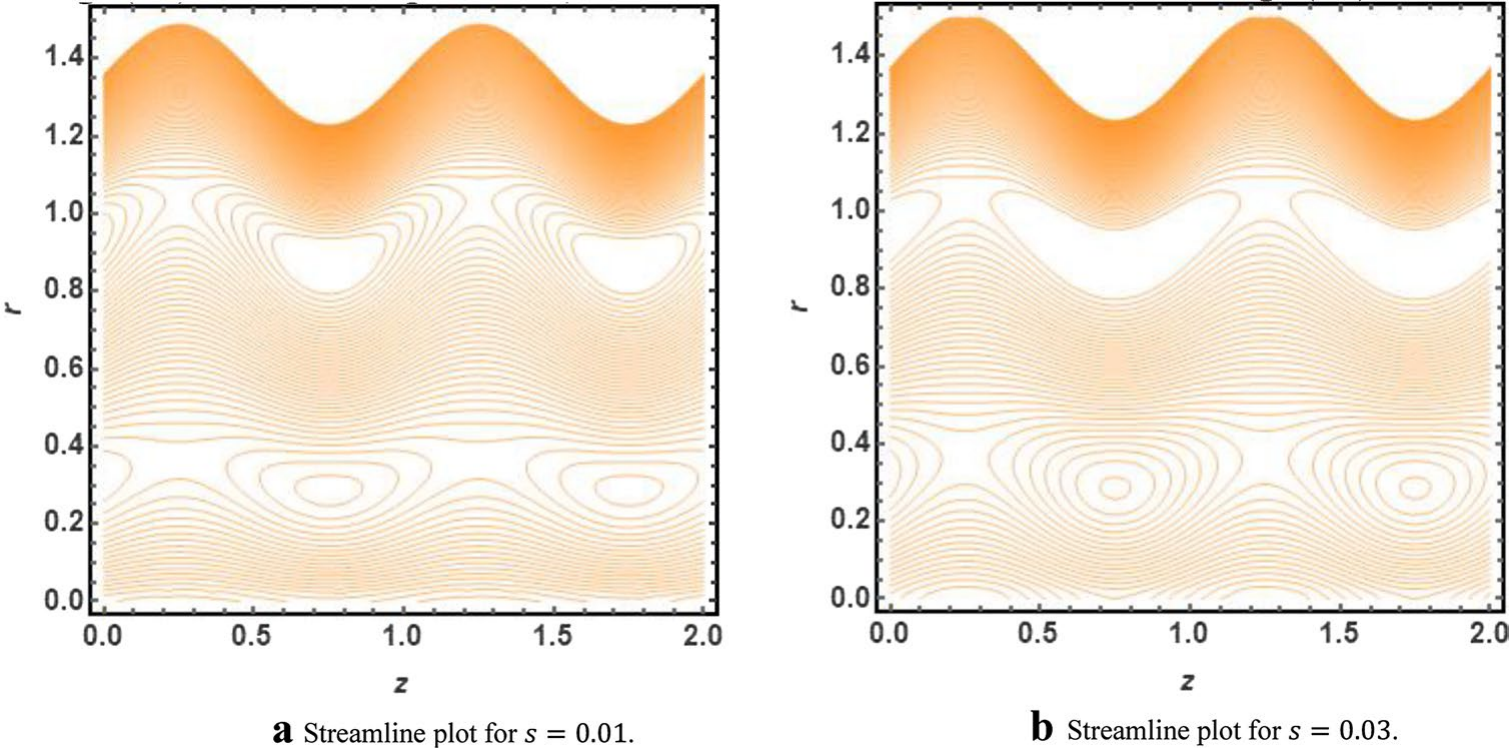
(**a**) Streamline plot for $s=0.01$. (**b**) Streamline plot for $s=0.03$.

The graphical illustration of above exact solution as well as entropy section is presented to verify the mathematical computations. The graphical solutions presented here clearly verify that the boundary conditions and flow profile are in accordance with the mathematical equations. The velocity profile of present problem is graphically presented by Fig. 2. Figure 2a shows that velocity declines with increasing value of $Q.$ Further, a fully evolved as well as parabolic profile that depicts maximum flow in the central region of these two sinusoidally fluctuating curved tubes and a decline in the flow profile is observed towards the boundaries. Figure 2b reveals the effect of increasing value of $s$ on the flow profile. The increase in the value of curvature parameter has an opposite effect on the flow profile at two different end walls (i.e. endoscopic wall and channel wall). Since the velocity declines with the inner endoscopic wall but the flow profile alters near the central region of these two curved tubes and velocity starts increasing near the outer wall of this curved channel for increasing value of $s$. The temperature graphical solutions of the present problem are conveyed through Fig. 3. The temperature plot for increasing ${B}_{r}$ is presented through Fig. 3a. A direct increase in the fluid’s temperature is noted with increasing ${B}_{r}$. It implies that viscous effects are prime reason for heat production in this case. Figure 3b unfolds that temperature is a decreasing function of $Q$. Since a decline in the heat generation is observed for incrementing values of $Q$. The effects of curvature parameter $s$ on the temperature profile are given through Fig. 3c. The generation of heat is increasing within the fluid for incrementing $s$. The pressure gradient’s graphical solution $\frac{dp}{dz}$ is plotted and provided by Fig. 4. Figure 4a conveys the graphical result of $\frac{dp}{dz}$ for increasing $Q$. It reveals that the value of $\frac{dp}{dz}$ is increasing with incrementing $Q$. The value of $\frac{dp}{dz}$ is also increasing with increasing value of curvature parameter $s$, as revealed in Fig. 4b. Figure 5a, b provide the graphical solution of $\Delta P$ plot against $Q$. A decline in the value of $\Delta P$ is observed for increasing $\phi$, depicted in Fig. 5a. Figure 5b unfolds an up-rise in the value of $\Delta P$ for increasing value of $s$. The graphical solutions of entropy $S$ against $r$ are presented through Fig. 6a, b. Figure 6a unfolds an up-rise in the total entropy of the system with increasing value of ${B}_{r}$. Since the increase in the value of ${B}_{r}$ eventually results in an up-rise of viscous effects that also increases the heat generation within the system, and it finally enhances the entropy. Figure 6b depicts the effects of $Q$ on entropy $S$. As the value of $Q$ increases, we have noted a declining entropy profile $S$. Entropy is minimum in the central region of these two curved tubes but it’s value increases near the walls of these two tubes. The minimum value of entropy in the centre is due to a fully evolved profile in the centre and there is no disturbance with the flow in centre but the sinusoidally deforming walls of both the peristaltic endoscope and the sinusoidal wall of outer tube are the reason behind this increased entropy near the boundaries. Figure 7a, b are plotted to analyze the effect of different parameters on Bejan number as well. Figure 7a coveys that ${B}_{e}$ increases with increasing value of ${B}_{r}$. Figure 7b also depicts an increment in the value of ${B}_{e}$ for increasing $Q$. The streamline plot is presented for increasing value of $Q$, as shown in Fig. 8a, b. An increment in the size of trapping is noted near the outer sinusoidal wall of curved tube but at the same time the size of trapping is declining near the inner sinusoidal wall of the peristaltic endoscope for increasing values of $Q$. Figure 9a, b present the streamline plot for increasing value of curvature parameter $s$. The number of trapped boluses are decreasing near the outer sinusoidal wall but the size of trapped bolus is increasing with incrementing $s$. The number of trapped boluses are increasing but it’s size is decreasing near the inner wall of peristaltic endoscope with increasing $s$.

## Conclusions

Entropy generation with combined evaluation of heat transfer is carried out for a novel peristaltic endoscope placed inside a curved tube having sinusoidally deformable walls. This is a new and advance topic related to endoscopic applications and certainly it will become a benchmark research problem for further interesting research work in this regard. Some of the major outcomes of this analysis are given as

- A parabolic and completely evolved flow profile is noted between these two sinusoidally fluctuating homocentric tubes.
- This novel peristaltic endoscope is more beneficial in endoscopy of human organs due to its flexibility and peristaltic locomotion.
- Many of the complex engineering structures and big machineries require such peristaltic endoscopes for their maintenance, since these peristaltic endoscopes can easily move within their structures to detect the problem.
- Entropy is nearly zero in the central region of these two curved tubes, (i.e. between this peristaltic endoscope and the outer wall of curved channel).
- Entropy starts increasing towards walls of the peristaltic endoscope and outer curved channel. Finally, becomes maximum near these walls.

## Data Availability

The authors states that all the files are provided in the paper no hidden file is required however if journal required any further data from us we will provide and the corresponding author is responsible to provide to the journal.

## List of symbols

${R}^{*}$(m): Radius of curved geometrical model
$$c$$ (m/s): Wave speed
$$\lambda$$ (m): Wavelength
$$s$$: Curvature parameter
$$\phi$$: Amplitude ratio
${r}_{1}$: Dimensionless radius of inner tube
${r}_{2}$: Dimensionless radius of outer tube
$\left(\overline{U },\overline{W }\right)(m/s)$: Dimensional form of velocity field
$$k$$ (W/m.K): Thermal conductivity
$${B}_{r}$$: Brinkman number
$${B}_{e}$$: Bejan number
$a$ (m): Mean radius of curved tube
$(\overline{R },\overline{Z })$ (m): Curvilinear coordinate system
$\mu$ (kg/m s): Dynamic viscosity
$b$ (m): Amplitude of peristaltic wave
$\beta$: Wave number in dimensionless form
${\overline{R} }_{1}$ (m): Dimensional radius of inner tube
${\overline{R} }_{2}$ (m): Dimensional radius of outer tube
$(u,w)$: Dimensionless form of velocity field
${C}_{p}$ (J/kg K): Heat Capacity
$$S$$: Dimensionless entropy
$\Omega$: Dimensionless temperature ratio
